# Epidemiology is ecosystem science

**DOI:** 10.1007/s11229-019-02129-5

**Published:** 2019-02-26

**Authors:** Keekok Lee

**Affiliations:** Faculty of Humanities, University of Manchester, Manchester, UK

**Keywords:** Ecosystem science, Epidemiology, Classical chinese medicine, Multifactorial causation, Non-linear causality

## Abstract

This paper primarily argues that Epidemiology is Ecosystem Science. It will not only explore this notion in detail but will also relate it to the argument that Classical Chinese Medicine was/is Ecosystem Science. Ecosystem Science (as instantiated by Epidemiology) and Ecosystem Science (as instantiated by Classical Chinese Medicine) share these characteristics: (a) they do not subscribe to the monogenic conception of disease; (b) they involve multi variables; (c) the model of causality presupposed is multi-factorial as well as non-linear.

## Introduction

This contribution explores the thesis that Epidemiology should be considered as Ecosystem Science; it does this through an examination of the following sub-themes.

1. Biomedicine exhibits two paradigms of explaining disease. The monogenic conception of disease, which is the dominant paradigm, embodies the standards and criteria of scientificity for Biomedicine. In contrast, Epidemiology is considered to be the Cinderella as its paradigm of explanation and scientificity is different and hence, held, at arm’s length, if not with outright suspicion.^1^
2. The two paradigms differ ontologically and methodologically. The monogenic conception of disease upholds the thesis: one causal agent, one disease entity. It rests on thing-ontology. Its implied model of causality is Humean, monofactorial and linear (the causal arrow is unidirectional, from cause to effect only). On the other hand, Epidemiology understands disease not so much as a disease entity but more as a pattern of interrelated events which may lead to a pattern of ill-health in the population. It rests on process-ontology. Its model of causality is non-Humean, multi-factorial and non-linear (the relationships are synergistic, reciprocal, with feedback loops).^2^
3. While the monogenic conception of disease is rooted in the Gold Standard of the Randomised Controlled Trial (RCT) and of late its related Gold Standard of Evidence-based Medicine (EBM), Epidemiology proceeds more in the manner of Ecology as a field science. Ecology explicitly studies ecosystems: the biotic and abiotic components which make up a particular ecosystem, the relationships between these with the ecosystem as a Whole, not to mention with other ecosystems. This kind of science is necessarily non-Reductionist as the Whole in terms of its causal inter- as well as intra-relationships are reciprocal in character; this complicated network of causal relations means that properties emerge from the Whole which cannot be predicted by simply adding up the contribution of each of the component parts of the Whole. Epidemiology is, hence, Ecosystem Science.^3^ It will also be shown that Ecosystem Science/Thinking itself may be considered as a variant of Systems Thinking.
4. If one cares to do some comparative history of science, the model of Ecosystem Science/Thinking and Systems Theory/Thinking can be said to be found in Classical Chinese *Medicine* (CCM for short, whose origin may be traced back to more than two and a half thousand or more years): CCM is a *science* which is *Wholist*^4^ in orientation, resting on process-ontology (rather than thing-ontology) and whose causal model is multi-factorial, non-linear and reciprocal, and with feedback loops.^5^
5. In a very brief extension of the theme at 4, this contribution looks at how CCM perceived/perceives epidemics and whether it had/has any grasp of what today we call Epidemiology. The conclusion is positive, although to mark the differences in spite of the similarities between CCM and modern Epidemiological thinking, the CCM discipline will occur in italicised form, namely, *Epidemiology*.

## The monogenic conception of disease, its paradigm of scientificity and its associated drawbacks

This conception is basically about infectious causal agents and how it has attained its prestigious status primarily through the work of two medical scientists, who are commonly recognised as intellectual giants, Pasteur (1822–1895) and Koch (1833–1910). Their research ushered in not simply the Age of Bacteriology but the era of “truly scientific medicine”. Pasteur is commonly acknowledged to be the father of the germ theory of disease which covers not merely harmful bacteria but also viruses. Koch was famous for his discovery of the tubercle bacillus as the cause of tuberculosis. Furthermore, he is associated with four methodological postulates which constitute the so-called Gold Standard for determining etiologically defined diseases in terms of infectious agents.^6^ Within two decades (1891–1899), germs were also found for cholera, diphtheria, typhoid, tetanus, plague, and rabies. This impressive array of discoveries put paid to previous theories of disease such as the miasma and the humour theories. The rise of the science of bacteriology also more or less coincided with the emergence of new therapeutic treatments, displacing traditional ones, such as venesection, generally recognised to be inefficacious. Pasteur developed a vaccine against rabies; Paul Ehrlich and Sahachiro Hata demonstrated that Salvarsan, an arsenical compound, could kill the spirochete of syphilis without drastic side effects, such as killing the patient. To be fair, one must point out that Koch’s vaccine against tuberculosis, tuberculin was a failure; an effective treatment did not appear until 1946 with the arrival of streptomycin. This, itself, heralded even the more impressive age of the antibiotic: Fleming had by chance discovered penicillin as early as 1928 but the mass manufacture of antibiotics was made possible through the efforts of Florey and Chain during the Second World War. As a result, antibiotics did not make a dramatic appearance until after the war.^7^

The rich theoretical crop of discoveries of infectious agents together with the new “magic bullet” of antibiotics has seared into the consciousness of the medical establishment as well as that of the lay public as a “golden age” in medicine and succeeded in putting Biomedicine upon an altar before which everyone bows low and deeply with the greatest awe and reverence. The Nobel Foundation in 1905 bestowed on Koch its award in medicine for his identification in 1882 of the tubercle bacillus as the cause of tuberculosis.

Koch also forcefully articulated the monogenic conception of disease in 1901. It suffices here to comment on only three aspects:

1. Koch said: “diseases are best controlled and understood by means of causes, in particular, by causes that are *natural*…”. At a stroke it destroyed the very old religious view that the cause of diseases was an expression of divine displeasure as well as its therapy in terms of prayer/miracle. Simultaneously, it also overturned the Hippocratic/Galenic account in terms of “fluid” medicine. “Solid” medicine became the new paradigm in medical thinking. A new research programme came into existence, a programme which after more than a 100 years is still regarded as “progressive” in spite of growing numbers of serious anomalies it has produced in its wake.
2. He said: “each disease is caused by one particular microbe—and by one alone. Only an anthrax microbe causes anthrax; only a typhoid microbe can cause typhoid fever”.^8^ One commentator has put it: “the final hope and aim of medical science is the establishment of monogenic disease entities (Taylor 1979, p. 21)”.
3. Koch strengthened the above by setting out his guidelines or methodological rules for ascertaining the cause which are sometimes called the Koch–Henle postulates:
  - The bacteria must be present in every case of the disease.
  - The bacteria must be isolated from the host with the disease and grown in pure culture.
  - The specific disease must be reproduced when a pure culture of the bacteria is inoculated into a healthy susceptible host.
  - The bacteria must be recoverable from the experimentally infected host.

Although these postulates have run into anomalies, nevertheless, they continue to be regarded as canonical, exercising a compelling hold over research in Biomedicine.^9^

Postulate 1 is key to understanding the monogenic conception of disease, as it holds that every disease has a single cause and that the cause is universal and necessary. This implies a notion of cause which is monofactorial and linear. This causal paradigm was simply borrowed from other successful Newtonian sciences such as physics and chemistry. It is said to be Humean which is also presented as the Billiard-ball account. This image is apt as it brings out certain features which are pertinent to the context of Biomedicine. The infectious-agent conception of disease as earlier remarked is “solid” medicine; the cause can be a bacterium/virus/fungus/poison. The cause, just like a billiard ball, is a solid object which can be seen (under specialised instruments such as the electron microscope, PET or TOM), collected and measured/quantified, or removed, and cultivated elsewhere outside the human body. Billiard balls left to themselves would not move without the introduction of an external force, that applied initially by the player via the billiard cue hitting the first billiard ball; analogously, the infectious agent is an external pathogen which invades the human body setting off a series of motions within.^10^

Although RCT and EBM constitute the Gold Standards of scientificity in Biomedicine as already observed, the former is not above challenge as it relies on randomisation to eliminate bias from its experimental set-up.^11^ This requires the trial to have primarily two arms, the experimental and the control. The former is given the treatment but not the latter. Should the difference in outcome between the two arms be positive (and also pass the test of being statistically significant), then this is attributed to the efficacy of the treatment. This conclusion appears to be methodologically sound only because the experimental set-up appears to conform to Mill’s experimental logic, that of the method of difference.^12^ RCT, relying, in the main, methodologically on the technique of randomisation amounts to presupposing the axiom which may be called the **axiom of homogeneity**. However, this axiom, in reality, fails to obtain except by resorting to the device of **deeming** participants/patients **to be homogenous**, save in one respect only. As a matter of fact, patients are **heterogenous**, not homogenous as a group. As a result, the RCT-EBM-approved-“efficacious” treatment may turn out to be irrelevant to their concern. Individual patients are not artefacts which can be ordered from a factory to conform uniformly to a very specific set of common characteristics. Randomisation as the key methodological/analytical technique can undoubtedly take care of allocation bias, but not other sorts of bias, such as selection bias. RCT’s inability to eliminate the latter type of bias renders its results at best statistically relevant and at worst clinically irrelevant. While hospital management in general and organisations such as NICE (in the UK) are interested in statistical relevance in respect of a drug’s efficacy, the “field” doctor who is concerned with individual patient care is interested in clinical relevance: a drug which passes the test of statistical relevance (via RCT-EBM) may be of no help to the doctor-and-her-patient, whereas a drug which has failed to pass such a test may be of help to the individual doctor-and-her-patient (given the patient’s own specificities of a medical-physical-psychological-social-moral kind known to the doctor who is in charge of her health).^13^ The **axiom of homogeneity** suffers from severe methodological limitations.

## Epidemiology and its paradigm of scientificity

Broadbent (2017, p. 93)^14^ characterises Epidemiology as follows:

Definitions of epidemiology vary, but include some common elements, especially the phrase “distribution and determinants of disease.” I define “epidemiology” as the study of the distribution and determinants of disease and other health states in human populations by means of group comparisons, for the purpose of improving population health. … Epidemiology is a discipline that essentially involves documenting the way health states occur in human populations, and trying to explain the documented patterns of occurrence. … Most epidemiologists, though not all, will also accept that the purpose of epidemiology is to improve population health. … the history of epidemiology definitely links it to both medicine and public health.

Epidemiology is, as confirmed by above, generally said not to be so much interested in the fate of individuals as patients but more in preventing the emergence of disease patterns amongst communities and populations. Certainly, the nineteenth-century pioneers such as John Snow (1813–1858, generally acknowledged to be the founding father of Epidemiology)^15^ during the cholera epidemic in London in 1854, was keen to work out why one neighbourhood in London suffered a cholera mortality rate 14 times greater than another neighbourhood, rather than investigate why this or that particular individual died of cholera. However, one should not misunderstand this to mean that successful epidemiological research would have no impact on individual lives as it clearly can and does—for instance, once the handle of the pump in Broad Street was removed, the death rate fell dramatically. While lab researchers concentrate on identifying the infectious agent and producing an effective form of treatment against the disease-causing agent, Epidemiology concentrates on public health measures to prevent a certain disease pattern from emerging.

The fall in the cholera mortality rate had nothing to do with the kind of knowledge celebrated by the monogenic conception of disease. Snow could only infer that there must be something unsavoury about that pump which made people fall ill upon drinking water contaminated by such a source. It was not till 1884, 30 years after Snow’s pioneering work in 1854, that Koch discovered that the infectious causal agent was the *vibrio cholera*.^16^

Time has moved on since the nineteenth century. Epidemiology, today, is said to be a young, developing science, whose “ancestry” is very recent indeed. Broadbent (2017, p. 93) writes:

Epidemiology did not emerge as a distinct field until the latter part of the twentieth century, and indeed teaching approaches and career paths are still not finalized. (Perhaps they will never be.)

The new pioneers may be said to be Austen Bradford Hill and Richard Doll whose research work is generally acknowledged to have put epidemiological investigations on an impeccable scientific footing from the methodological point of view. (Doll’s substantial findings cover not only the tobacco-lung-carcinoma link, but also between other substances such as asbestos and cancer, radiation and leukaemia, alcohol and breast cancer as well as establishing that smoking increases the risk of heart disease). Their work in establishing that tobacco is a crucial factor in the production of lung cancer led to the banning of smoking in public space and other measures to discourage smoking.

**Fig. 1:**
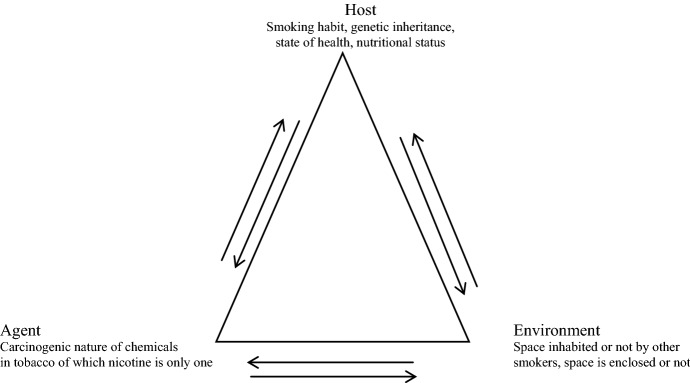
Epidemiological (enhanced) triangle of relevant variables and causation

The causal model invoked by this kind of epidemiological research (in the context of tobacco smoking and lung cancer) implies a more sophisticated variant of Epidemiological Thinking which may be represented by the image of the Triangle of Causation,^17^ but greatly enhanced, as shown below (Fig. 1).

There are three main variables: Host, Agent and Environment between which the causal arrows indicate reciprocity. This advanced causal model obviously differs from that exhibited by the monogenic conception of disease (and, indeed, from the earlier Snow model of Epidemiological Thinking). Below is a quick comparison between the two models.

**Table:** 

	Monogenic, linear	Epidemiological, non-linear
	a	b
I	Humean/Billiard-ball	Non-Humean
II	Monofactorial	Multi-factorial
III	One cause, one effect	Inter-acting causal variables leading to even a synergistic effect^18^
IV	Causal direction moves in a single uni-directional straight line	Causal direction is reciprocal, from A to B, B to A^19^
V	Static, ahistorical	Dynamic, historical
VI	Atomistic materialism: the whole is no more than the sum of its parts	Wholism: the whole differs from/is greater than the sum of its parts; emergent properties
VII	Reductionist	Non-reductionist
VIII	Solid medicine/thing-ontology	Patterns of events in populations/process-ontology

Note that in the smoking-lung cancer example not only is it the case that there are three main variables involved in the model of causation, but that each of these three main variables is internally complex.^20^

*Host* includes several variables, such as the smoking and/or the alcohol drinking habits of the individuals, their respective genetic inheritances, general state of their health/age/nutritional status/occupational status.

*Agent* includes the carcinogenic nature of not only one chemical, but the many chemicals found in tobacco smoke, of which nicotine is only one.

*Environment* includes whether the space in which the individuals dwell/work consists of smokers, even if they themselves do not smoke, whether the space is enclosed or not, and if enclosed whether adequate ventilation obtains, and if not enclosed, whether the air outside is polluted or clean, and so on.

Of late some researchers appear to want to develop this more refined characterisation of Epidemiology Thinking even further, to approximate it closer to what this contribution calls Ecosystem Science, which Systems Thinking claims also to encompass. For instance, O’Connor and McDermott (1997) distinguish a system from a heap: the former is a series of inter-connected parts which function as a Whole; when parts are removed or added to, it changes; its behaviour depends on its overall structure (the arrangements of its component parts). The latter is a mere collection of its parts; its parts function independently of one another; hence the arrangement of its components as well as overall structure is irrelevant, as its behaviour depends merely on its size. The notion of system can be used to describe complex biological relationships (such as in ecology/study of ecosystems). It may be used to characterise relationships/processes found in an organisation. It may also be used in understanding illnesses, their diagnosis and treatments as found in CCM (see section to follow on the subject).^21^

**Fig. 2:**
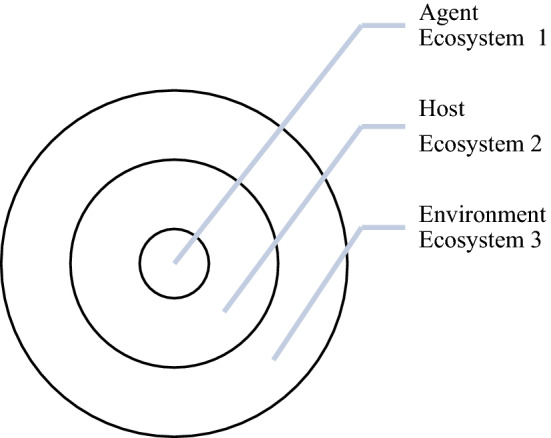
Epidemiological causation as ecosystem nesting of concentric circles

Ecosystem Thinking/Systems Thinking is keen to point out that the relationship between events/processes involves complicated systemic linkages, drawing out clearly such a methodological implication of Wholism. One can re-cast the enhanced Triangle of Causation in terms of nesting ecosystems: the Host (the person) as Ecosystem 1, the Agent (the chemicals in tobacco smoke which are carcinogenic to the Host) as Ecosystem 2, the Environment as Ecosystem 3. Indeed, 1, 2 and 3 may be said to form another more-encompassing ecosystem, namely, Ecosystem 4. See Fig. 2 below.

The most important aspect of Systems Thinking which have impressed researchers working at the cutting edge of Epidemiological Thinking is that “from a systems perspective, health is conceptualized as an emergent property of a system, in which processes operating at the levels of individuals and populations are inextricably connected” (Diez Roux 2011). Systems approaches emphasise the need to understand dynamic interrelations between various components.

Because the effect of a given input depends on other conditions in the system, emphasis shifts from isolating the causal effect of a single factor to comprehending the functioning of the system as a whole. Complex systems typically include heterogeneous agents at various levels, contact structures between agents, adaptation, nonlinear dynamics, and stochasticity. These features lead to the emergence of patterns at various scales. (Diez Roux 2011)

Such an approach involves identifying and studying the presence of important causal loops in the system. In many types of population health problems feedback mechanisms are found between behavioural and environmental features as well as between health and social circumstances. An instance of the former is this: the availability of healthy food promotes a healthier diet which in turn creates an increase in the demand for healthy foods; an instance of the latter is: health affects income and income in turn affects health. These are self-reinforcing tendencies.

To enlarge a little. Take the case of incidence of smoking, and ultimately of its related problems of disease and ill-health and attempts to lower it. Admittedly each of the following interventions produces some effect, each in its own right: smoking cessation programs, prohibiting smoking in public spaces, increasing cigarette taxes, social marketing and so on. However, the Epidemiologist in accordance with Systems Thinking would also consider what would happen when all the relevant interventions are combined; the total joint effect might be greater than the sum of their effects when produced in isolation from one another (synergistic effects). In a system, when changes in a factor/variable provide feedback into the process, generating feedback loops which may produce positive or negative impacts. Suppose high taxes on cigarettes would reduce the number of smokers. The next step to pursue is to see if higher taxation would lead to changes over time in social attitudes to smoking which may then make it possible to increase enforcement of public smoking regulations. If it does, then this would be a positive feedback impact. On the other hand, raising cigarette taxes would mean the treasury has less money to render smoking cessation programs accessible to help individuals to quit the habit. This then would lead to a negative feedback impact.

In other words, Systems Thinking embodies a Wholism whose processes constitute a dynamic system with very intricate feedback loops which can reinforce the processes of change or dampen them. Under the former, a new equilibrium would eventually be reached; under the latter, equilibrium would be restored. Wholism accommodates both instability and stability; both positive and negative feedback loops can occur in a system.

With that brief outline about Systems Thinking in place, let us then look at Ecology as post-Newtonian science whose key concept of the ecosystem has given rise “eponymously” to what may be called Ecosystem Science/Ecosystem Thinking, which emphasises the importance of feedback loops (both negative and positive) in the history of ecosystems.

## Ecology: ecosystems and ecosystem science

The discipline called Ecology became accepted as a science in the way understood today only after the Second World War, especially through the work of Eugene Odum.^22^ It may be defined as that branch of science which studies the relations and interactions between organisms and their abiotic environments as well as among the organisms themselves, at the level of communities, populations, ecosystems both at local and global scales. An ecological community may be defined (albeit simplistically) in terms of a group of actually or potentially interacting species of organisms living in the same place—one well known type of possible interaction is of course the prey-predator relationship, another is mutualism. A population in ecological literature refers to a group of individuals of the same species inhabiting the same area—for instance, the Arctic supports a population of polar bears. Ecology concentrates on population rather than on the individual organism in a population; one reason amongst others is that scientists/society have neither the economic nor technological resources to study individual organisms, even very large ones, like large mammals such as polar bears. Furthermore, their primary focus is on characteristics of population: distribution (the area in which the population exists); abundance or size of the population; its rates of growth, birth and death; spacing and dispersion, emigration and immigration (as animals are mobile, seeds of plants can spread via wind, water, mobile animals), predator–prey relationship; disease which can affect population growth either directly by killing off the young before they can even reach reproductive age or by affecting the reproductively mature animal through undermining their health and, thereby, their reproductive performance.^23^

Ecology studies ecosystems. The notion of an ecosystem may be spelt out in some detail as follows:

1. Ecosystems do not come labelled as such; scientific investigators have to identify and delimit them for the purpose of their study at hand. However, this does not mean, that they are abstract theoretical entities with no bearing to reality on the ground. Some have clearly identifiable boundaries (such as a meadow, an estuary), others are not so obviously the case. Some can be very big, such as Antarctica or the Arctic, others infinitesimally small in comparison, such as a handful of soil.
2. Any ecosystem, such as that of Yellowstone Park or the Gobi Desert, occupies a certain portion of time and space which has a historical dimension both in terms of time and space. For instance, neither Yellowstone nor the Gobi had existed from time immemorial, nor historically would the space they each commanded be necessarily identical to that they each now respectively occupy. It is sometimes possible, though not frequently, to ascertain the precise beginning of an ecosystem, such as the sudden throwing up of a small island in the middle of an ocean as a result of an undersea volcanic eruption.
3. An ecosystem evolves, changing all the time—at time t_1_, a particular item predominates, at time t_2_, that item may have retreated somewhat, its predominant position taken over by another item. For instance, in a shoreline ecosystem at t_1_, large cliffs stand out, but at t_2_, some of the cliffs might have collapsed into the sea below, generating a new landscape with changes for the ecosystem involved, even to the extent of perhaps creating two related but different ecosystems.
4. Overtime, one ecosystem may evolve into a distinctively different ecosystem. For instance, take a lake. Of all the geological formations, lakes are said to be the most ephemeral as they evolve and change the quickest. A lake would shrink (for a variety of reasons, climate change, either of a local or global kind, another could be excessive extraction of water from it by humans), and would dry out completely, eventually evolving to become a meadow. Inland seas too shrink such as the ongoing shrinkage of the Aral Sea.

(On points 2, 3 and 4, refer back to point Vb which contrasts with Va in section above.)

5. An ecosystem may be defined in terms of all the organisms of each species living in community interacting with other communities as well as with all the abiotic factors in their habitat. As far as the latter is concerned, the dynamics of an ecosystem involve three key abiotic processes which cannot be caught simply by population processes and phenomena: these are energy flow, hydraulic flow, and chemical cycling with which the population interacts. In an ecosystem, the complex interplay between the numerous variables operating within it determines the characteristics of the population in question.

(See points II b, IIIb and IVb which contrast with IIa, IIIa and IVa in section above.)

6. Every ecosystem is necessarily an open system. It is in principle an open system as ultimately it requires input from outside Earth, that is to say, from our Sun, as we have seen, to supply it with sunlight (energy) to maintain it. It cannot violate the laws of thermodynamics.
7. Every ecosystem must be grasped as a Whole; an ecosystem Whole cannot be understood in a reductionist manner. In other words, this Whole has properties which are over and above, not simply the sum of the properties of all the constituent parts. Let us briefly look at this implication of Ecosystem Wholism by considering a handful of soil. The soil consists of both biotic (usually micro-organisms but also macro-organisms such as worms which are visible to the naked eye) as well as abiotic elements, such as water, air, chemicals (PH content, etc.). The character of the soil (such as its texture) cannot be accounted for solely in terms of the properties of each of its constituent components—it may be said, therefore, to be an emergent property of the Whole system, born of the complex interplay between all the variables involved in that handful of soil and the micro-climate of which it is a part.

(See points VIb and VIIb which contrast with VIa and VIIa in section above.)

8. This complex inter-play may be displayed through considering a hypothetical potential creation of an ecosystem as follows: first, a hair-line crack occurs in a rock, conceivably produced by the difference in temperature between day and night, summer and winter (A); next, water (B) enters the crack, turning into ice in the winter, thereby enlarging the crack in the process; a seed in the autumn floats by, lodges itself in the crack and the following spring begins to grow (C); C together with B cause A to widen, which in turn permits more water and ice to enter, giving more space for C to grow by widening the crack still further and thereby allowing more rain and frost (B) to enter and erode it, and so on. These processes of change and development show that the causal paradigm at work is dynamic, historical, reciprocal (with positive feedback), synergistic, multi-factorial, non-linear.

(See points Ib, IIb, IIIb, IVb, Vb which contrast with points Ia, IIa, IIIa, IVa and Va.)

9. An ecosystem may display negative feedback loops—the prey-predator relationship is one such example. Imagine the population of foxes increasing in a particular habitat. Foxes prey on rabbits. Increase of foxes would mean decrease of rabbits, but as rabbits are eaten up by foxes, the predator would suffer from a shortage of prey. As a result, the fox population would decline; the rabbit population would instead increase. As a result, equilibrium is restored between predator and prey, equivalent to a negative feedback loop. It is seen to be at work in regulating populations in ecology. An ecosystem would be de-stabilised if its populations (whether between animals and animals, plants and animals, plants and plants) exceed its carrying capacity.
10. An ecosystem also exhibits positive feedback loops which are responsible for the sudden appearance of rapid changes. Positive feedback involves a circular set of effects which are self-reinforcing. For instance, an ecosystem primarily of grasses with few shrubs may be stable for five to ten or more years. Yet such an ecosystem may change over time and the change would occur within a relatively short period of time. Shrubs which started off by being few and far between amongst the grasses, take a much longer time than grasses to establish themselves in the ecosystem, as their roots go deeper and initially competed unfavourably for rain water with grasses, whose roots are in the topsoil. Hence the ecosystem remains basically a grass ecosystem. However, over time, the shrubs, having established themselves, albeit slowly, would begin to grow taller, overshadowing the grass. Then seemingly suddenly, the grasses would be at a disadvantage in the competition for sunlight, fade and not flourish as well as they have done in the past. The grass ecosystem would have then turned itself into a shrub ecosystem. As established shrubs are more successful in capturing the available water and sunlight than the grasses, these will decrease dramatically. Positive feedback loops act as forces of change and are a source of instability.

This brief sketch of Ecosystem Wholism makes obvious a very important ontological point: it focuses not so much on things (biotic and abiotic), as on relationships between them which involve events and processes. Ecosystem Thinking occurs within the framework of what may, then, be called process-ontology whereas the thinking behind the monogenic conception of disease occurs within the framework of thing-ontology. The latter (monogenic conception of disease) differs profoundly from the former (Epidemiological Thinking) which may be said to be a type of Ecological Thinking. Things are the objects of study by Newtonian Science; these are macroscopic objects, with definite boundaries, varying in actual size from a planet/heavenly body to microbes and atoms. Things/macro-sized objects possess the following characteristics: they are visible (if not to the naked eye, then with the help of instruments), touchable, impenetrable, have form, shape and size, and are measurable. On the other hand, post-Newtonian sciences (such as Ecology and Epidemiology) involve process-ontology and the rejection of Humean linear causality, as already observed.

(See points Ib, VIIb and VIIIb which contrast with Ia, VIIa and VIIIa in section above.)

In a nutshell, the succinct differences between thing-ontology/substance-ontology and process-ontology may be spelt out in the words of Rescher (1997, p. 2) as follows:

Process metaphysics as a general line of approach holds that physical existence is at bottom processual; that processes rather than things best represent the phenomena that we encounter in the natural world about us. The doctrine takes a position within the spectrum of competing following contentions:

1. Process has *primacy* over things. Substance is subordinate to process: Things are simply constellations of processes.
2. Process has *priority* over substance. Things are always subordinate to processes because processes inwardly engender, determine, and characterize the things there are. But processes as such transcend the realm of things since there are also substance-detached processes.
3. Substance has *priority* over process. The only sort of processes…are (sic) those involved in the doings and comportment of things.
4. Substance has primacy over process. Indeed, substance is all there is; all processes and changes are simply a matter of how things appear to certain (mind-equipped) substances.

The first two of these competing contentions represent process philosophy respectively in its stronger (Heraclitean) and weaker (Empedoclean) versions. By contrast, the substance approach which process philosophy rejects is represented by the last two contentions. This approach also has a weaker (Democritean) and a stronger (Parmenidean) version.

(See point VIIIb in contrast to point VIIIa in section above.)

In the history of Western philosophy, the dominant metaphysics is thing-ontology; hence, it is not a surprise that Newtonian sciences are based on that ontology. Process-ontology made a brief appearance in ancient Greek philosophy, primarily in the fragments of thought left by Heraclitus; Leibniz (eighteenth century) is considered to be the next contributor. However, it is only in the twentieth century that process- philosophy has been systematically articulated in the later writings of Whitehead; and even then, it has not been taken up by scientists, only by some theologians. In quantum physics, Bohr implicitly introduced process-ontology via his notion of complementarity; but Bohr was not so much influenced by Leibniz as by ancient Chinese *philosophy*, in particular by the *Laozi* which is Daoist or *Daojia philosophy*.^24^ On the other hand, Bohm (1976, p. 40), the physicist-cum-philosopher has written:

In this movement, there is NO Thing. Rather, things are abstracted out of the movement in our perception and thought, and any such abstraction fits the real movement only up to a point, and without limits. Some ‘things’ may last for a very long time and are fairly stable, while others are ephemeral as the shapes abstracted in perceptions of clouds.

## Classical Chinese *medicine* is ecosystem science

Lee (2017a, 2018) demonstrate that ancient Chinese *philosophy*, as found in *Daojia philosophy* (whose foundational texts include the *Laozi*, the *Zhuangzi* and the *Huainanzi*, just to mention three) rests on process-ontology, based on *Qi* as the fundamental ontological category (operating under two modes, *Qi*-in-concentrating mode as thing and *Qi*-in-dissipating mode as process/processes with the latter being *primus inter pares* in respect of the former^25^). Such a *philosophy* endorses or implicitly uses for its *science* a causal model which is multi-factorial, non-linear and reciprocal. CCM as a pre-eminent *science* whose metaphysical/methodological core can be traced back to its roots in *Daojia philosophy*, is clearly an instance of what this author calls Ecosystem Science/*Scienc*e. As Ecosystem *Science*, it is *Wholist* in its ontology and non-Reductionist in its methodology.^26^

We can present CCM’s Ecosystem Thinking in terms of concentric circles as Ecosystem-nesting.

**Fig. 3:**
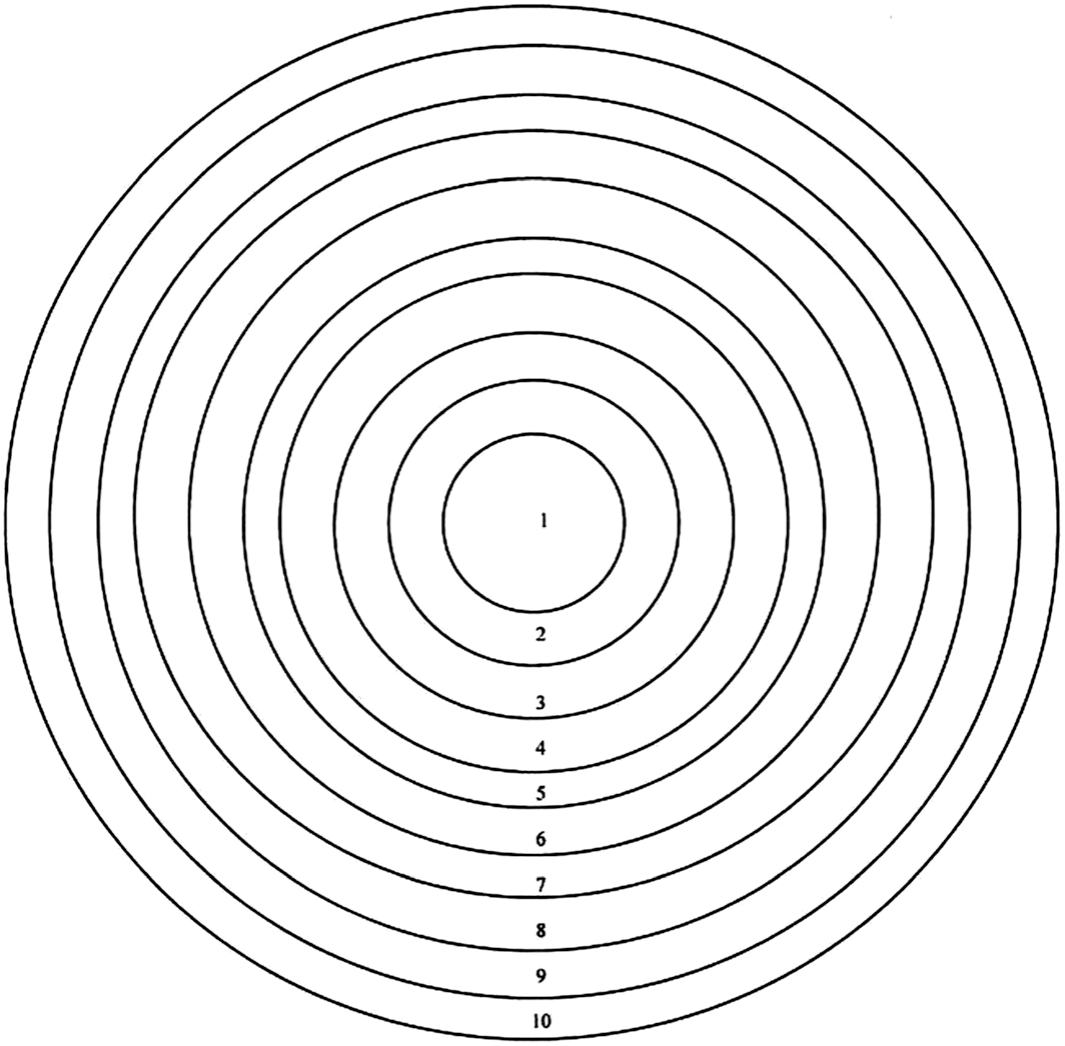
Ecosystem-nesting in terms of concentric circles. 1 Cell, 2 Tissue, 3 Organ-system, such as the **Spleen-stomach**/脾胃organ-system, 4 All visceral organ-systems (*Wuzang*-*liufu/*五脏六腑), 5 Entire material parts and total functioning of the person including emotions, 6 *Qi* in *yuzhou* (Macrocosm) as well the *Jingmai* via the *Jingluo* network of the person-body (Microcosm), 7 Immediate external environment, in which a person lives (air, water, food, shelter, climate….), 8 Social/cultural environment (tribes/ethnic groups/polity), 9 Larger physical/social environment, in which person lives (plants/animals/rivers), 10 Cosmological environment, in which a person lives (Sun/Moon/Earth…)

Although Fig. 3 shows ten concentric circles, it is important to point out that CCM is not interested in circle 1 but in the remaining nine circles marked 2-10. These circles may be called *Ecosystems*. Also note that whereas in Biomedicine, the spleen and the stomach are regarded as two different/separate organs, in CCM, the **Spleen-Stomach** constitutes a single organ-system (***Ecosystem 3***), and all the five organ-systems constitute in turn a larger ecosystem (***Ecosystem 4***); this is because although different and separate as anatomical entities (or things), in terms of their *physiological* functioning (as events/processes), they are intimately intertwined. To illustrate this point in greater detail, take *Ecosystem 3*: it is about the relationship between the *yin* organ and the *yang* organ, such that each visceral organ-system/*Zangfu*/脏腑 has a *yin* as well as a *yang* component. For example, the **Spleen** (*yin*) pairs with the **Stomach** (*yang*) as *piwei*/脾胃, the **Heart** (*yin*) with the **Small Intestines** (*yang*), the **Lungs** (*yin*) with the **Large Intestines** (*yang*), the **Liver** (*yin*) with the **Gall-bladder** (*yang*), the **Kidneys** (*yin*) with the **Bladder** (*yang*). This ***Ecosystem*** is then an instantiation in CCM of *Yinyang Wholism*, a fundamental *Wholism*. As already observed, the *yin* organ is called *Zang*/脏, and the *yang* organ *Fu*/腑. Together, they are often called *Wuzang*-*liufu*/五脏六腑. It suffices here to point out that in Chinese numerology, *wu*/five, being an odd number, is *yang* in character, while *liu*/six, being an even number, is *yin* in character; therefore they also form part of *Yinyang Wholism* as ***Ecosystem 3***.

*Ecosystem 5* indicates that for CCM, the concept of person is a primitive one, that is, the person embodies both physical and mental/emotional characteristics or what may be called Mind–Body *Wholism*, implying the rejection of Mind–Body dualism which underpins modern Western philosophy (since Descartes) and, in turn, Biomedicine (resting on Body-Mind dualism). It also implies that for CCM, all illnesses have, in principle, a psychosomatic dimension and that treatment must take this dimension into account.^27^

*Ecosystem 8* reinforces the point made by ***Ecosystems 5*** by emphasising that individual psychology is not itself independent of the value system of the individual’s community/society.

*Ecosystems 7 and 10* embody another larger *Wholism* within which the individual person is embedded. *Qi* in the individual person (Microcosm) is affected by *Qi* in the universe (Macrocosm).^28^

*Ecosystem 9* indicates that the individual person, her community/society are all part and parcel of another *Whole*, namely, the greater physical/natural environment within which the community/society lives and works.

*Ecosystem 3 and 4* As far as illness is concerned, if the *yin* organ is affected, its *yang* counterpart may also be affected, and vice versa. If one visceral organ-system is affected, it is likely that, sooner or later, another organ-system would also be affected, as all members of this greater *Whole* are governed by *Yinyang*–*Wuxing*.^29^

*Wuxing*’s five phases are:

**Table:** 

**Water**	*yinqi* reaches its maximum and *yangqi* its minimum
**Wood**	*yinqi* retreats/decreases while *yangqi* begins to advance/increase
**Fire**	*yangqi* reaches its maximum
**Earth**	*yinqi* and *yangqi* are at equilibrium
**Metal**	*yangqi* retreats/decreases while *yinqi* begins to advance/increase

*Wuxing* invokes two main modes of operation: the Mutually Engendering and the Mutually Constraining. Let us focus first on the former. **Water** stands for **Winter** (when *yinqi* is at its maximum) but as water is an indispensable requirement for Life and Life’s activities, **Water** is said to generate **Wood**. **Wood** stands for **Spring** (when *yinqi* begins to retreat and *yangqi* to rise) and therefore for all Life and its activities. **Wood** in turn is said to engender **Fire** (as it is obvious that to start a fire one would need a fuel such as wood, and **Fire** stands for **Summer** when *yangqi* is at its maximum). In turn **Fire** engenders **Earth**; **Earth** permits organisms to thrive but when these die, they decay and are returned to **Earth** itself, or when wood burns, all that is left is its ashes which become soil). **Earth** stands for a period when *yinqi* and *yangqi* are in equilibrium. **Earth** engenders **Metal** (in the sense that **Earth** harbours both the biotic and the abiotic, including metals in its bowels). **Metal** stands for **Autumn** (when *yangqi* begins its retreat as *yinqi* rises, and as the ancient Chinese noticed that when the Moon was full, the tide was high, and they were particularly impressed by the high tide at the time of the full moon during the eighth lunar month of their calendar, which marks the beginning of **Autumn)**. **Metal/Autumn** generates **Water/Winter** (as *yangqi* decreases even further until it reaches its minimum and *yinqi* reaches its maximum).

We next look at the Mutually Constraining mode. The ancient Chinese clearly knew that vegetation was vital to prevent soil erosion and soil loss, especially during heavy rains when exposed soil with no vegetal cover could be washed away; by growing trees on such surfaces, soil erosion would be prevented. So in this sense, they postulated that **Wood constrains Earth**. Heavy rains could cause floods; to prevent flooding, one could use earth to build dykes and dams—in this sense, the ancient Chinese postulated that **Earth constrains Water**. To prevent a fire from spreading, one would need water to damp down the fire—in this sense, it could be said that **Water constrains Fire**. To make implements such as an axe, a sword, one would need fire to melt the metal before one could cast or mould it—in this sense, the ancient Chinese held that **Fire constrains Metal**. An axe (made from metal) would be useful in chopping down trees which in turn would provide wood to fuel fires—in this sense, the ancient Chinese postulated that **Metal constrains Wood**.

**Fig. 4:**
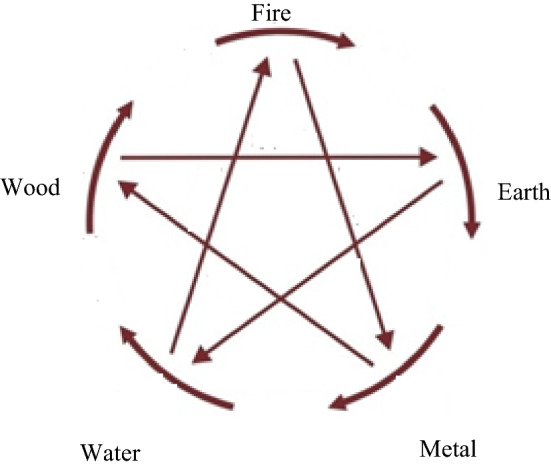
The thick broken lines of the circle and their arrows stand for the mutually engendering cycle while the thinner unbroken lines and their arrows inside the circle stand for the mutually constraining cycle

While the above explication may be helpful, one should not forget that *Wuxing* is really about the relationship between *yinqi* and *yangqi* in the course of a year (or of a day). A normal relationship means that in **Winter**, *yinqi* is at its maximum and *yangqi* is at its minimum, and that in **Summer**, the reverse relationship obtains. However, illness occurs when the relationship is not normal for the time of year in the individual person as ascertained by feeling the *mai* (normally translated as pulse but which this author thinks should be resisted). But in this context, let us understand *Wuxing* not so much in CCM but in general in the context of applying it to explain ecological phenomena. Abnormal relationships between *yangqi* and *yinqi* are mediated by two sub-concepts called Deficiency (*buzu*) and Excess (*taiguo*). We see these concepts at work clearly in the Mutually Constraining as well as the Mutually Engendering modes. Look at Fig. 4 again and, in particular, at **Fire** constraining **Metal**. When **Fire** is in excess, this means that **Metal** would be over-constrained by **Fire**; such over-constraining would provoke **Metal** to “fight back” not, however, directly against **Fire**, but against **Water** instead, as **Metal** engenders **Water**. One could then say that **Fire** has, ultimately, “met its own come-uppance” or achieved an “own goal”, as **Water** constrains **Fire**. Let us call this Example A.

Using the same instance of **Fire**, let us see what happens when its *qi* is in Deficiency—(let us call this Example B). This would result in **Water** taking advantage of such a weakness to damage/undermine **Fire** as well as in **Metal** reacting by “insulting” **Fire**, leading ultimately in this chain of reactions to **Earth** being undermined, as **Fire** engenders **Earth**. Spelt out a bit more fully, it runs as follows: **Water** constrains **Fire or Fire** is constrained by **Water**. When **Fire** *qi* is deficient, **Fire** would be unduly constrained by **Water** and thus, **Water** would undermine/damage **Fire**. **Fire** constrains **Metal** or **Metal** is being constrained by **Fire**. However, as **Fire** itself is undermined and weakened by **Water**, **Metal**, in reaction, would take advantage of **Fire**’s weakness and turn round to undermine **Fire**. In the **Engendering** cycle, we know that **Fire** engenders **Earth**; but here **Fire** itself has been weakened and its weakened state would in turn impact upon its process of engendering **Earth. Earth** constrains **Water,** but weakened Earth would let **Water** *qi* be in excess. **Water** *qi* in excess, in constraining **Fire**, would weaken **Fire** even more.

The post-Newtonian sciences of today such as cybernetics, systems theory, ecology and others are said typically to display feedback mechanisms, both negative and positive. The section above have already cited examples of both negative and positive feedback loops in Ecological Thinking. The prey-predator relationship has been cited as an instance of the former; here we can use it to illustrate the relevance of an aspect of *Wuxing* Thinking. Cast in the language of *Wuxing*, it would run as follows: Excess of **Wood** *qi* (increase in fox population) would over-constrain **Earth** *qi*, resulting in decrease in the rabbit population; this leads to Deficiency of **Earth** *qi*, which in a state of humiliation would hit back “insulting” the stronger party, resulting in a decline in the fox population.

Example B could be illustrated by considering the relationship between vegetation, earth/soil and Water. One can present it as follows: Cutting down trees excessively causes soil erosion/loss; less soil brings about less regeneration of tree growth; fewer trees cause greater loss of water/moisture in the soil; more soil loss/erosion … This positive feedback loop which involves a vicious (rather than a virtuous) circle in the language of *Wuxing* could be presented as follows: **Metal** constrains **Wood**. When excessive tree cutting occurs, **Wood** would be adversely affected (with fewer trees left standing). **Wood** constrains **Earth**; with fewer trees around, **Wood**’s ability to constrain **Earth** would result in Deficiency (in other words, soil loss/erosion would increase). **Earth** constrains **Water**, but with soil loss/erosion, **Earth** can no longer perform adequately the role of holding back water, owing to its *qi* Deficiency. **Water** engenders **Wood**; but when **Earth** can no longer constrain **Water**, **Water** in turn, in this context, would result in Deficiency, and **Water** would be unable to engender **Wood**. When **Water**’s capability of engendering **Wood** is adversely affected, the end result of this chain of *Wuxing* reactions would be an even greater loss/erosion in soil than the initial loss.^30^

## Chinese *Epidemiological* thinking is necessarily ecosystem thinking

The section above has set out in very brief outline the claim that CCM is Ecosystem Science. CCM, ever since its beginnings, has suffered no paradigm shift in its *philosophical* (and hence also methodological) foundation.^31^ Its *philosophical*-cum-methodological framework has also consistently embraced all domains in which ill-health manifested/manifests itself both at the level of the individual who fell/falls ill or at the population level when whole swathes of people fell/fall ill such as during epidemics.^32^ In these respects, CCM differs from Western Medicine as Western Medicine has suffered a rupture between Greek/Galenic medicine and modern medicine (see Text box 1 in following section) on the one hand as well as, on the other hand, a paradigm shift from the monogenic conception of disease-entity (presupposing thing-ontology and a linear, monofactorial model of causality) to Epidemiology which this author has argued embodies Ecosystem Thinking (presupposing process-ontology and non-linear, multi-factorial model of causality).

Like all societies, Chinese society in its long history has suffered epidemics from time to time. The ancient Chinese referred to epidemics as *dayi*/大疫, *ji·yi*/疾疫 or *li*/疠 amongst others. These include in today’s nosological categories from the Chinese *medical* standpoint *wenyi*/瘟疫 (febrile epidemics^33^), *liuxing ganmao*/流行性感冒 (influenza), *mafeng bing*/麻风病 (leprosy).^34^ The earliest occurrence of *ji·yi*/疾疫 or *dayi*/大疫 recorded in Chinese history was during the Zhou dynasty (1046-256 BCE). The number of occasions throughout the various dynasties from the Zhou to the Qing dynasties is listed as: Zhou, 1; Qin-Han (221 BCE-220 CE),13; Wei-Jin (220–420 CE), 17; Sui-Tang (581–906 CE) 17; Song (960–1279 CE), 32; Yuan (1279–1368 CE) 20; Ming (1368–1644 CE), 64; Qing (1644–1912), 74.

Let us take a look at Zhang Zhongjing/张仲景 (150–219 CE), a practitioner-cum-theorist in the late Han dynasty, ranked in the history of Chinese *Medicine* as just below the Yellow Emperor who is commonly said to have had a hand in writing, if not considered to be the sole author of the foundational text, the *Huangdi neijing*/*The Inner Canon of the Yellow Emperor*. Zhang Zhongjing probably completed his book, the *Shanghanzabinglun/*《伤寒杂病论》/*Discourse on Cold Damage and Other Illnesses*, a few years after 200 CE.^35^ During his lifetime, Sima Qian in the *Historical Records*/*Shiji* mentioned 22 natural disasters—drought, floods, landslides, earth-quakes, locusts, famines, dykes bursting. It also coincided with a long period of unrest, with continuous wars, including those before the Three Kingdoms period (220–265 CE) as well as those occurring during it. This meant that the economy suffered, production went down. The conjuncture of natural disasters, economic chaos and war would inevitably lead to all sorts of unimaginable social ills. People were driven from the land, and therefore, into poverty, hunger, illnesses, and even cannibalism, with death and corpses everywhere throughout China’s heartland. Under such circumstances, epidemics naturally flourished. Zhang Zhongjing wrote, in the Preface to his book, that of his own extended family/clan which included more than 200 people, two-thirds had died; 70% of those, who perished, had died from the epidemic raging then from 196 CE. Historians have calculated that roughly half of the Chinese population could have perished in total. As a result, Zhang Zhongjing was not only determined to help relieve suffering but also seize the opportunity to study the epidemic, to collect as much information as he could, including the prescriptions which were used by physicians at large to handle effectively some of the cases involved.^36^

No suspicion, naturally, would be cast on the appearance of epidemics in Chinese history; but the sceptical may immediately doubt that the ancient Chinese might have the concept of epidemiology at all, even if the ancient concept would not be as sophisticated as the one which Broadbent (cited earlier) has written about. The quick way to quell this doubt has already been just given, namely, that as CCM suffered/suffers no rupture of any kind, it follows that it embodied/embodies Ecosystem Thinking and such Ecosystem Thinking would cover all domains of its theory and practice. In other words, this means that in treating the phenomenon called epidemics, CCM would have used that very framework which Epidemiology today (this paper argues) deploys. This, in turn, would mean that CCM would have grasped the concept of epidemiology whether one deigns to use the term “epidemiology” or not; but as is the wont of this author, the term would be used but italicised, namely, it would appear as *Epidemiology* to mark both the similarities as well as the differences between them. What is further needed to add force to this general argument is to talk briefly about how the ancient Chinese physicians had recognised features which were/are distinctive of epidemics in order to cope adequately with the phenomenon of epidemics itself.

Two strategies immediately come to mind, deployed by the ancient Chinese physicians to cope with epidemics, which are relevant to the preoccupation here. These were: (a) the proper disposal of the dead to prevent further spread of the epidemic; (b) quarantine to contain the epidemic. They implied/imply that the physicians understood that illness in an epidemic could be a contagious matter, that is to say, that such an illness would spread to more and more people via direct contact with those already afflicted. The preparation for the burial of those who had died from it and the presence of corpses which no one in the larger family or even clan could bury as families and clans themselves were dead or dying, was a task left to the state. The first record of such intervention occurred during the pre Qin–Han period—in the *Zhouli*/《周礼》,^37^ the abandoned dead were interred by state authorities. After this, the strategy became standard procedure during epidemics, with the state buying the coffins and carrying out the whole process of preparing the body for internment. By Song times, the state had resorted to getting Buddhist monks to carry out this task, and rewarded those who performed it with a special licence to preach their religion. In 1208 CE, the Song state instituted another policy: to encourage volunteers to bury the dead; anyone who buried two hundred abandoned bodies (who were mainly amongst the poor in society) would be given a reward (presumably financial). Furthermore, from the Northern Song onwards, the government also set up designated public sites where the poor could bury their dead and abandoned bodies could be interred. This policy greatly curtailed the spread of an epidemic.

We next look at the strategy of quarantining those affected. The first recorded attempt is found in the Han dynasty, in the *Hanshu/History of the Han Dynasty*, in the chapter about the reign of the Emperor Ping/《汉书·平帝纪》. The *Hanshu* is attributed to the historian Ban Gu/班古 (and various members of his family). The passage reads: 元始二年,旱蝗,民疾疫者,舍空邸第,为置医药 which may be rendered (by this author) as: “In 2 CE, a plague of locusts ravaged the land, followed by an epidemic which swept through the population. The government of the Western Han dynasty (206 BCE–9 CE) made available residences (usually occupied by the aristocracy or high officials) to house the afflicted (as a form of quarantine) as well as to treat them.” By the time of the Northern and Southern Dynasties (386–589 CE), quarantine had become routinized—during the *Xiaoqi* period (420–581 CE), a crown prince (太子长懋) and others established six specialist quarantine quarters.

In the Tang dynasty, the government mainly used Buddhist monks to organise medical aid for beggars who became ill, to house the afflicted in quarantine quarters. In the Song period, the state ran on a big scale homes for the afflicted which doubled up as quarantine establishments. An example of this occurred in 1076 CE; in Yuezhou/越 州, in the spring of that year, an epidemic occurred to which the government responded by building a large home/hospital for the ill and to quarantine the afflicted. (See Chapter 19 of《越州赵公救灾记》/*The Records of Lord Zhao’s Disaster Rescue Attempt*.) In 1089 CE, an official famous for his poetry, Su Shi/苏轼 in Hangzhou also set up similar accommodation. The practice became entrenched in government policy in the dynasties which followed, although it must be observed that its expansion was not the norm, instead sometimes, it even shrank. The vacuum in many instances was filled by charitable bodies.

Apart from providing accommodation for the purpose of quarantine, during the Jin dynasty/晋代 (265–420 CE), its archives recorded (《晋书.王彪之传》) the following edict in 356 CE: “朝臣家有时疫, 染易三人以上者,身虽无疫, 百日不得入宫” which is rendered (by this author) as: “Should three members in any family of the court officials be affected by the epidemic, it is forbidden for such officials to attend court for a 100 days.” The epidemic referred to here was leprosy, a highly contagious illness which was rampant in earlier times.^38^

The two strategies set out briefly above are especially relevant to any attempt to throw light on the understanding of the nature of epidemics on the part of the ancient Chinese physicians and on whether they had any grasp at all of what we call Epidemiology today for the following reasons:

1. An epidemic in the medical context is commonly defined in dictionaries as: A widespread occurrence of an infectious disease in a community at a particular time (see Oxford English Dictionary). The ancient Chinese use of *dayi* or *jiyi* satisfies this dictionary sense in English.
2. The ancient Chinese physicians (and the imperial officials) implicitly recognised that an epidemic could involve an illness which was infectious but which could also be an illness called contagious in today’s medical language; hence to stop the spread of the epidemic, they realised that one must dispose of the victims properly.
3. While the ancient Chinese physicians did not abandon, as we have seen above, the use of medicinals to help individual victims, nevertheless, they realised that the best strategy was isolation, hence quarantine.
4. Contagious illnesses necessarily involved/involve a community, a population. In other words, the best strategy, namely 3 above really implied/implies that CCM had an implicit grasp of the concept of epidemiology as their strategies appear to be concerned with “the distribution and determinants of disease”.
5. The ancient Chinese physicians were aware of the determinants of illness in an epidemic because: (a) they had observed that not only the features already mentioned above were relevant, but that since very early times, they knew that an external pathogenic factor was involved. In the late Shang dynasty (1600–1046 BCE), the Oracle Bone script referred to *chong/*虫, *gu*/蛊, *nüe·ji*/疟疾^39^ as some of the possible causes of illnesses which if not contagious would have been infectious; (b) CCM was/is very clear that adverse factors internal to the person’s constitution together with adverse factors in the external environment, such as droughts, floods, unseasonal heat/cold, earthquakes, sudden environmental changes including wars which disrupted/disrupt production leading to famine and hunger played/play key role in the emergence of illnesses afflicting large swathes of the population. All these variables acting together (between a and b) could bring about an epidemic, as their mode of thinking, this paper has argued, was/is Ecosystem Thinking.
6. As CCM is Ecosystem Science, it was/is well aware that any human population must live within bigger ecosystems which include the greater natural environment, that is, *Ecosystems* 6, 7, 9 and 10 set out in Fig. 3 are all involved.
7. For the reasons set out briefly above, it may be appropriate to say that CCM had/has some grasp of the concept of epidemiology, even though its version might not be identical with modern Epidemiology. Hence it might be appropriate to refer to it as Chinese *Epidemiology*.
8. Apart from the two looked at above, a third relevant strategy needs some brief consideration. This pertains to that of containing the devastating effects of smallpox/*tianhua*/天花, which had evolved and developed from the long-held CCM idea of using toxin to fight toxin. Its first successful use was recorded in 1653. This may be cited as evidence for *Epidemiological* thinking in CCM. The strategy consists of infecting persons with a mild form of smallpox which would not kill them but instead render them immune to the illness in all its normal ferocities. CCM talked about cultivating different types of attenuated smallpox (such as *shuimiao*/水苗, *hanmaio*/旱苗) for the purpose. Following news of success, the Japanese and the Russians introduced the strategy into their countries. The Turks also came to know about the new treatment of inoculation/variolation as Lady Mary Montague, wife of the British ambassador to the Ottoman Court in Istanbul learnt about it and in 1717, she successfully used it on her own children. When she returned to Britain in 1721, she introduced the idea/technique to the Royal College of Physicians who naturally rejected it; but she persuaded Princess Caroline to test it. Small scale trials were carried out, in one case involving seven death-row convicts at Newgate prison who were offered reprieve provided they agreed to take part in the trial which they did and all survived. However, later trials showed that one in eight of the treated died of the very disease they were being protected against; this still, however, compared favourably with the going rate of about 30 percent of the disease’s untreated victims. This paved the way for Jenner (1749–1823)’s vaccination technique. The point here is not to trace the historic link between the Chinese and the Western attempts at immunology, but simply to say that the former’s attempt implied/implies a grasp on the part of CCM physicians that a contagious illness such as smallpox could in principle be contained.^40^ Vaccination today is a recognised tool in Epidemiology.
9. So far the discussion has only touched on Chinese *Epidemiology* which covered/covers^41^ infectious/contagious illnesses. However, Epidemiology today is much less concerned with contagious disease-entities, at least in First World economies (since the introduction of antibiotics at the end of the WWII) than with diseases such as obesity, high blood pressure, heart problems, etc. The “distribution and determinants” of such diseases are, on the whole, said by Epidemiology to be caused by inappropriate/unhealthy diets/lifestyle, and that the way forward must be to wean people away from such unwholesome to more wholesome ways of living. Did the ancient Chinese physicians have a grasp of such issues? The short answer is yes, via their general concept of *Preventive Medicine* which encompasses the more specific concepts of *yangsheng*/养生 or *yangshen*/养身and of *shiyang*/食养. The former refers to how to live, nurture, nourish yourself both physically and spiritually in order to lead a long and healthy life; the latter, how to eat properly in order to maintain a long and healthy life. (For details, see Lee 2018, Chapter 4: *Preventive Medicine* (Primary Meaning) in the Context of CCM as Ecosystem Thinking; Chapter 8: *Fang* and *Food*.) In other words, Epidemiology and *Epidemiology* share this same basic goal and roughly the same way forward in achieving that goal.

## Overview of the ontological and methodological differences between Newtonian and post-/non-Newtonian medicine/science

The text box below summarises the main ontological and methodological differences between medicine/*medicine* as Newtonian Science and non- or post-Newtonian Science.^42^

**Table:** Text box 1 Differences between Newtonian and non- or post-Newtonian science/medicine

	BM (monogenic conception)	CCM (including *Epidemiology*) and epidemiology
	a	b
I	Solid medicine	Changing patterns of *yinqi* and *yangqi* (*Qi*-in-dissipating mode and *Qi*-in-concentrating mode) in the case of CCM only but not in the case of epidemiology
II	Atomistic materialism; the whole is no more than the sum of its parts	*Em*-*ism* in the case of CCM only; *Wholism/*Wholism: the *Whole/*Whole differs from/is greater than the sum of its parts; emergent properties
III	Static, ahistorical	Dynamic, historical
IV	Reductionist	Non-reductionist
V	Linearity	Non-linearity
VI	Humean/Billiard-ball bounded by Newton’s Laws of motion	Non-Humean, outside the domain of Newton’s Laws of Motion
VII	Monofactorial	Multi-factorial
VIII	One cause, one effect	Inter-acting causal variables leading to synergistic effects
IX	Causal direction in a single uni-directional straight line: →	Causal direction is reciprocal, from A to B, B to A: ↔; negative and positive feedback loops
X	Incompatible with ecosystem thinking	Ecosystem science/ecosystem thinking
XI	Treating the individual patient	Treating the individual patient *Wholistically* in CCM but also pertinently in *Epidemioloy/*epidemiology focussing on patterns of illness/disease in populations
XII	Thing-ontology	Process-ontology
XIII	Newtonian science/medicine	Non-/post-Newtonian science/medicine

## Conclusion

This contribution has tried to set out the following theses:

1. The dominant conception of disease in Biomedicine even today is the monogenic conception implying its hallowed paradigm of scientificity in terms of the twin Gold Standards of RCT and EBM. Although Biomedicine has an impressive list of achievements to its name, and remains a still progressive research programme, nevertheless, it has run into anomalies. Furthermore, it is a case of doing science within the Newtonian framework of thing-ontology and whose model of causality is Humean, monofactorial and linear (the Billiard-ball model).
2. In contrast, Epidemiology is perceived to lack the “kudos” of the monogenic conception, as it departs from the paradigm of scientificity of its senior partner. For instance, RCT is irrelevant to its methodology. It focuses on disease population patterns rather than on disease-bearing individuals who succumb to diseases. It rests on process-ontology (events/processes); the enhanced/sophisticated versions of its causal model are multi-factorial, non-linear (with feedback loops), reciprocal and synergistic. Instead of relying on drugs or surgery as treatment, Epidemiology relies on public health measures such as ensuring water and air sanitation, discouraging certain lifestyle habits (such as smoking or consuming too much sugar).
3. Ecology is Ecosystem Science/Thinking and is a variant of Systems Theory/Thinking whose philosophical framework Epidemiology Thinking appears to share both in terms of ontology and the model of causality which its Wholist orientation implies. Ecology, too, studies populations rather than individual organisms. They may all be regarded as post- or non-Newtonian sciences.
4. Although all three (Systems Thinking, Ecology and Epidemiology) are pioneering non-Newtonian sciences (beginning in the twentieth century), in the long *durée* of human civilisation, their philosophical presuppositions when made explicit can be said to have a history of at least two and a half thousand years. This is for two reasons: (a) CCM is an instantiation of ancient Chinese *Science* and it has an uninterrupted history of at least two and a half thousand years; (b) CCM, too, rests on process-ontology, and instantiates a causal model which is multi-factorial, non-linear and reciprocal. At all levels of theory-and-practice, of organisation and analysis, it is *Wholist* in orientation and is fiercely non-Reductionist in its methodology.

On the subject of CCM and Systems Thinking, Hejazi (2013) writes:

11. Have there been cultures that have wholistic systems thinking?

Yes, there are. In Chinese culture everything is comprehended within the conceptual frame of Yin and Yang that is a holistic system. Yin and Yang are counterparts for negative and positive or female and male. Yin and yang are co-dependent. Within such a system, the notion of absolute independence makes no sense. A human relationship is also a state of interdependence; both extreme dependence and independence are seen as dysfunctional. Yin and yang also complete each other; neither is fully realized without the other. For humans, full individuality is realized in the context of the other. And when yin and yang are combined together, we get the Tao, a synergistic emergence and co-creation; something new emerges that transcends the parts.

However, this should not be taken to mean that Chinese *Science* and CCM as well as Chinese *philosophy* underpinning them do not differ from post-Newtonian sciences such as Ecology/Ecosystem Science, Systems Theory/Thinking and Epidemiology in several other ways. It might not be out of place to remind readers yet again of such differences which undoubtedly exist, although not emphasised here; to mark them, terms such as “*Wholism/Wholist*”, “*Science*”, “*Medicine*”, “*Epidemiology”* and “*philosophy*” are italicised when used in the Chinese context.

5. Based on the above comments and arguments, this contribution feels justified to come to its main conclusion that Epidemiology (today) could be considered as Ecosystem Science/Ecosystem Thinking, and hence, of Systems Theory/Systems Thinking.
6. Furthermore, based on the historical evidence found in Chinese history in general and CCM canonical texts in particular, one may plausibly conclude that CCM down the ages had/have an understanding of epidemics and a grasp of some of the key concepts of epidemiology. However, similarities apart, there are obvious differences between the ancient Chinese understanding, on the one hand and modern understanding on the other. To mark both the similarities as well as the differences, it seems fitting to cast the CCM version in italicised form as *Epidemiology* and to refer to the modern form as Epidemiology.

## Footnotes

1 See Lee (2012).

2 See Lee (2017a).

3 See McIntosh (1985), Meadows (2009), Ehrlich et al. (1977). On the notion of emergence in general, see Mitchell (2012), O’Connor and Wong (2012).

4 I deliberately use the terms “Wholist” and “Wholism” rather than the more normal “holist” and “holism” in order to emphasise that ontologically speaking, the Whole is more than the sum of its component parts. Further to distinguish between Ecology and Epidemiology on the one hand and CCM on the other, and to mark the differences between them, terms such as “*Wholism/Wholist”* and “*science*” are italicised in the latter but not in the former.

5 See Lee (2017a, 2018).

6 See Thagard (2000). See also Lee (2012), Chapters 9 and 10 for a more detailed discussion and critique.

7 See Tansey (1997).

8 As cited by Evans (1993, p. 20).

9 Warren and Marshall were awarded the Nobel Prize in Medicine and Physiology in 2005 (on the hundredth anniversary of the prize to Koch) for their discovery that *Helicobacter pylori* is the cause of peptic ulcer; for a critique of their work and of Koch’s postulates in general, see Lee (2012, pp. 98–103 and 108–110).

10 The latest addition to this model of solid medicine within the Billiard-ball causal framework is genetic material with the emergence of DNA genetics and molecular biology since the late 1950 s. Although not an external pathogen, the cause is, nevertheless, a piece of matter (a snippet of DNA) which could be either snipped off or inserted in order to eliminate a disease in the individual. This model neither departs from thing-ontology nor from monofactorial causation even though in reality, so far at least, it applies only in the rare cases of diseases identified as caused by single-gene defects.

11 As EBM involves a meta-analysis of RCTs, any methodological flaws which may be found in RCTs will necessarily undermine the soundness in turn of EBM.

12 See Mill (1843); for details of this interpretation of Mill, see Lee (2017b, 2018, Chapter 7).

13 For detailed arguments of points raised above, see Lee (2017b).

14 See also Bhopal (2008, p. 3) for his much shorter definition; according to Riegelman and Kirkwood (2015, p. 232): “Our definition of population health—the totality of all evidence-based public and private efforts to preserve and promote health and prevent disease, disability, and death. This broad twenty-first century definition requires public health agencies and professionals to collaborate with a range of government agencies and healthcare professionals and institutions”.

15 In fact, a little earlier in 1847, Ignaz Semmelweiss (1818–1865) had carried out a type of investigation, methodologically similar to that of Snow. He demonstrated that puerperal fever was infectious as well as contagious and that its incidence was significantly reduced when medical staff were instructed to wash their hands, after visiting the mortuary and touching the cadavers, before tending to patients in the maternity ward.

16 The truth is more complicated. Nobody, including Koch himself, knew that the discovery had already been made in 1854 by an Italian scientist called Filippo Pacini (1812–1883). Pacino died poor and obscure. In 1965, 82 years after his death, the international committee on nomenclature adopted *Vibrio cholera Pacini 1854* as the correct name of the cholera-causing organism. See UCLA (2017).

17 See Bhopal (2008, pp. 131–135).

18 In general, it may be defined as an effect which obtains when several variables interact with one another leading to an outcome that is greater than the sum of individual effects arising from each of the variables in isolation. See Lee (2017a, pp. 305–309).

19 See Dickens and Flynn (2001), Lee (2017a, p. 304).

20 This is what is meant by “multi-factorial” in this context. (When Broadbent talks about “multifactorialism”, he could be referring to plural causes, that is, an effect brought about, independently, by more than one cause. For instance, peptic ulcer can be caused by either *Helicobacter pylori* or the over use of NSAIDS.).

21 See Hejazi (2013), Lee (2017a, pp. 21, 126–132, 138, 311, 325).

22 For a biography, see Barrett (2005); for the 5th edition of his iconic book, see Odum and Barrett (2005).

23 On these points, see Berryman and Kindlmann (2008).

24 See Lee (2017a, c).

25 *Qi*-in-concentrating mode sooner or later returns to *Qi*-in-dissipating mode. In terms of the modern science of thermodynamics, one says that a thing sooner or later disintegrates as entropy increases; in this process, sometimes, heat/energy is released when, for example, a piece of coal is burnt eventually to ashes while letting off heat as it burns. For a detailed discussion of *Qi* as the fundamental ontological category and of the distinction between its two main modes of operation, see Lee (2017a).

26 This account of CCM is necessarily brief and selective as its aim here is simply to present CCM as Ecosystem Thinking/Systems Thinking and as *Ecosystem Science*. For detailed exploration, see Lee (2017a, 2018).

27 For readers interested in detailed textual and philosophical exploration of this set of topics pertaining to *Ecosystem 5*, see Lee (2018, Chapter 6) which also touches on the relationship between *Wuxing, Wuzang-Liufu* and *Wuqing* (the basic five emotions).

28 For a detailed exploration of the relationship between *Ecosystems* 6, 9 and 10, see Lee (2017a, Chapters 7, 10 and 11).

29 *Wuxing* literally means five phases/five processual phases and is but an aspect of *Yinyang*; it spells out these transformative-phases of *Yinyang* when the *yinqi* changes in relationship with the *yangqi* during the yearly cycle of the seasons or the daily cycle of night-day. *Qi* exists either predominantly as *yinqi* or as *yangqi*. For details, see Lee (2017a, Chapter 7).

30 Needham and Wang (1956, p. 258) is correct in observing that ecology provides good examples to illustrate *Wuxing* at work.

31 It is important to note that this author distinguishes CCM from what today is called Traditional Chinese *Medicine* (TCM). The former has been practised uninterruptedly for more than two and a half thousand years while the latter could at best be said to have existed for only seventy. For a detailed exploration of the theoretical and other differences between them see Lee (2018, Chapter 11).

32 Note that in the context of CCM, it is more appropriate talk about “illness/illnesses”, “being/falling ill” rather than “diseases”, “suffering from disease”, as the term “disease” in Biomedical thinking today refers to “disease-entity”; such thinking and language presuppose thing-ontology.

33 This translation is found in Hanson (2011), Introduction, 170 (endnote 3). She has also written: Today the Chinese “Warm Diseases” category includes the afflictions biomedicine class as acute infectious diseases. They are also febrile diseases due to a climate-sensitive external pathogen that causes one’s temperature to rise and fever symptoms to set in. The most virulent and contagious forms of *wenbing* become epidemics. (2011, p. 165).

34 *Mafeng* literally means “numbing wind”. In ancient *medical* texts, the term referred to many skin conditions including those which in today’s Biomedical diagnostic terms is leprosy—see Leung (1999, 2009).

35 For a detailed account of the vagaries of his life and his work and an appreciation of his contribution to the development of CCM, see Lee (2018), “Appendix 2”.

36 See *Hao (2011), Lecture One.

37 A book mentioned as being in the collection of Old Texts in the library of the Han dynasty prince, Liu De/刘德 who died in 130 BCE. Chinese scholarship claims it to be a work compiled during the Warring States period (see ChinaKnowledge 2018).

38 For the historical information cited in this section, see **Zhongyi shijia*/中医世家 (2018); *Li (2016), where further details are found.

39 The first two mentioned refer in general to (harmful) insects, worms, bugs, while the second refers to exceptionally lethal organisms. Hanson (2011, p. 7) says it refers to magical poisoning, as practised by shamans. However, there could be more to *gu* than meets the eye, although this is not the place to go into details. For the more limited purpose in hand, one could say it was a method of nurturing and nourishing certain venomous organisms, such as snakes, spiders, centipedes to concentrate their poison either as a weapon for seeing off one’s enemies or under certain circumstances even as a form of medical therapy.) The third referred to what today we would identify in certain contexts as malaria – 疟 in general refers to “intermittent fevers”.

40 This account is complicated by the fact that alternative claims exist. For instance, that variolation actually originated in Turkey (although Ottoman texts did not mention it at all) or in India; yet another, that the Welsh had practised it since 1600 (this however, was oral evidence from one person only)—see Boylston (2012). For detailed references on the part of Chinese *Epidemiology* and *Immunology* in the history of CCM with regard to smallpox, see *Cao Dongyi and Zhu Shengjun (2007); see also *Leung (1987–1988).

41 The SARS episode involved the use of techniques which CCM on the whole would have approved, although the intervention was conducted in the main under the aegis of TCM—see Hanson (2010), Lee (2018, Chapter 9).

42 For thorough exploration of these key concepts, see Lee (2012) (for BM), 2017a, 2018 (for CCM).
